# Costs and cost-effectiveness of different follow-up schedules for detection of occupational hepatitis C virus infection

**DOI:** 10.1136/gut.2007.145516

**Published:** 2008-09-29

**Authors:** S Deuffic-Burban, D Abiteboul, F Lot, M Branger, E Bouvet, Y Yazdanpanah

**Affiliations:** 1 CTRS INSERM U795, Lille, France; 2 LEM-CNRS, Lille, France; 3 Groupe d’Etude sur le Risque d’Exposition au Sang (GERES), Paris, France; 4 Hôpital Bichat-Claude Bernard, Paris, France; 5 Institut de Veille Sanitaire (InVS), Saint Maurice, France; 6 Service des Maladies Infectieuses et du Voyageur, Centre Hospitalier de Tourcoing, Tourcoing, France; 7 EA 2694, Faculté de Médecine de Lille, Lille, France

## Abstract

**Objective:**

The purpose of this study was to compare the costs and cost-effectiveness (C/E) of early hepatitis C virus (HCV) RNA testing (alternative-US recommendations) after occupational exposure to HCV with existing follow-up strategies: (1) French, anti-HCV antibodies and alanine transaminase (ALT) activity at months 1, 3 and 6; (2) European, monthly ALT activity for 4 months and anti-HCV antibodies at month 6; (3) and baseline-US, anti-HCV antibodies and ALT activity at month 6.

**Methods:**

A decision tree simulated each strategy for 7300 healthcare workers (HCWs) exposed to HCV each year in France, taking into account the impact of early diagnosis on the response to antiviral treatment and the deterioration of HCW quality of life after exposure.

**Results:**

For a HCV transmission risk of 0.5% after exposure, the French strategy led to the highest costs/person (€181.40) and the baseline-US strategy to the lowest (€126.60) (€178.50) for alternative-US). The shortest mean time to HCV infection diagnosis (1 month) and the lowest number of chronic hepatitis C (CHC) patients (1.9/7300 HCWs exposed) was obtained with the alternative-US strategy (vs 6 months and 7.9 CHC, respectively with baseline-US). Compared with the alternative-US, the French strategy was associated with higher costs and lower utilities, and the European with a higher incremental C/E ratio. Compared with the baseline-US strategy, the alternative-US strategy C/E ratio was €2020 per quality-adjusted life year saved.

**Conclusion:**

In HCWs exposed to HCV, a strategy based on early HCV RNA testing shortens the period during which the HCW’s wait for his HCV status, leads to lower risk of progression to CHC and is reasonably cost-effective.

Infection by hepatitis C virus (HCV) is an important occupational hazard for healthcare workers (HCWs). In France, in 2004, 41 276 accidental blood exposures occurred in hospitals, 58.7% of which were percutaneous injuries.^1^ Of these cases, 6.2% occurred to anti-HCV antibody-positive source patients and 24.1% to source patients whose status was not known but who were considered as potentially anti-HCV antibody positive. In the USA, 1 385 280 sharp injuries were estimated to occur annually, and 22 000 HCWs were exposed to at least one percutaneous injury with a sharp object contaminated with HCV^2^ (http://www.who.int/quantifying_ehimpacts/publications/en/sharps.pdf).

Different follow-up schedules for occupational HCV detection have been recommended in different countries. Whereas European guidelines recommend alanine transaminase (ALT) monitoring alone,^3^ French and baseline-US guidelines are based on anti-HCV antibody and ALT monitoring,^4^ ^5^ but alternative-US guidelines propose HCV RNA testing at 4–6 weeks if earlier diagnosis of HCV infection is desired. The risk of transmission is first determined by the status of the source patient: when the occupational exposure involves an HCV RNA-negative source patient, the risk is considered to be zero; when the source patient is HCV RNA-positive, it is estimated at 0–10.3%,^6^^–^16 with an average rate of 0.5%.^11^ ^17^ The results of a recent case–control study conducted in five European countries suggested that after occupational exposure to HCV, assessment of the risk of transmission should take into account the severity of the injury and the device involved.^18^ Our hypothesis is that follow-up schedules should be tailored to the HCW’s risk of HCV seroconversion after percutaneous exposure.

In this study, using a cost-effectiveness (C/E) analysis, we compared the three existing follow-up strategies recommended in France, Europe and the USA after occupational exposure to HCV, with a strategy based on early HCV RNA testing, according to the risk of HCV transmission.

## MATERIALS AND METHODS

### Study design

We designed a decision analysis model to compare in HCWs exposed to an HCV-positive source the three existing strategies of follow-up and a strategy based on early HCV RNA testing:

- Strategy 1: monitoring of anti-HCV antibodies and ALT activity at months 1, 3 and 6 after HCV exposure, and HCV RNA testing to confirm positive anti-HCV antibody results and/or ALT elevation (French recommendations).^4^
- Strategy 2: monthly monitoring of ALT activity for 4 months after HCV exposure, and of anti-HCV antibodies at month 6, and HCV RNA testing to confirm ALT elevation or positive anti-HCV antibody results (European recommendations).^3^
- Strategy 3: anti-HCV antibody and ALT activity monitoring at month 6 after HCV exposure, and HCV RNA testing to confirm positive anti-HCV antibody results (baseline-US recommendations).^5^
- Strategy 4: HCV RNA testing 1 month after HCV exposure (alternative-US recommendations), as proposed by US recommendations if earlier diagnosis of HCV is desired.^5^

Baseline anti-HCV antibodies and ALT activity were determined, but, as they were identical for the four strategies, they were not taken into account in this analysis. When the HCV RNA test was negative during follow-up, a second HCV RNA test was requested to confirm the absence of HCV infection, because undetectable HCV RNA at one time point does not exclude the possibility of HCV infection.^19^ ^20^ When the HCV RNA test was positive, a second HCV RNA test was required before treatment administration.

The present model-based study simulated the trajectory of 7300 HCV-seronegative HCWs (ie, number of HCWs percutaneously exposed to anti-HCV antibody-positive source patients each year in France),^1^ who were followed from exposure to death. Model outcomes included the number of patients who developed chronic hepatitis C (CHC), number of incidents of CHC avoided by early therapy, lifetime quality-adjusted life years (QALYs), direct medical costs and incremental C/E ratios. The incremental C/E ratio was defined as the additional cost of a specific strategy compared with the next least expensive strategy, divided by its additional clinical benefit.^21^ We adopted a societal perspective considering everyone affected by the intervention and counting all significant health outcomes and costs that flow from it from the time of intervention to death.^21^ Future costs and clinical benefits were discounted at 3% per year.^21^ Costs were expressed in 2006 Euros (€1.00 = US$1.34 = £0.78, 17 October 2008).

### Model structure

#### Follow-up strategies after occupational exposure

The decision tree simulated four follow-up strategies after occupational exposures to HCV (Supplementary fig 1). The trajectory of HCWs through each strategy is described in detail in the legend of Supplementary fig 1. In brief, we modelled the trajectory of the HCWs in such a way that we were first able to estimate, based on the sensitivity of tests used for each strategy, the time to HCV positivity detection for that strategy. Next, in those in whom HCV transmission occurred, we were able to model the probability of hepatitis C spontaneous resolution, the benefit of treating acute hepatitis C earlier in the absence of the spontaneous resolution of the infection and the course of CHC disease in the absence of a response to acute hepatitis C treatment.

#### Follow-up in the case where a treatment for acute hepatitis C was initiated

Treatment for acute hepatitis C was administered for a duration of 6 months.^22^ A positive HCV RNA test at the end of treatment corresponded to non-response to treatment and the patient was considered to have CHC. For those with a negative HCV RNA test at the end of treatment, HCV RNA was tested again 6 months later. A positive test at that time corresponded to a relapse and the patient was considered to have CHC, whereas a negative test corresponded to a sustained viral response (SVR). The follow-up of treatment for acute hepatitis C consisted of three outpatients visits and three ALT measurements at 1, 3 and 6 months after the start of therapy, one HCV RNA test at the end of treatment and one 6 months later.

#### CHC disease course

Life expectancy, health-related quality of life and lifetime costs of patients with CHC were estimated from the study of San Miguel *et al*^23^ where the course of HCV disease was modelled in a cohort of Spanish patients with CHC, with a median age of 42 years, who had previously not responded to interferon (IFN). We used a study in which the course of patients who had not responded to antiviral therapy was modelled because in our analysis all HCWs with CHC had failed a first antiviral treatment for acute hepatitis C. In the study of San Miguel *et al*, patients received an additional course of standard IFN and ribavirin combination for 12 months and the course of the disease was simulated until death.

### Input data

#### HCV transmission probability

In the base case analysis, the average risk of HCV transmission after occupational exposure considered was 0.5%.^11^ ^17^ In the sensitivity analysis, a lower and a higher risk were considered. We used data from a recent case–control study of risk factors for HCV transmission to estimate the risk of HCV transmission after occupational exposure according to the presence of one or several risk factors.^18^ We found that in the most favourable cases, superficial injuries corresponded to a low risk of 0.009%, whereas, in the least favourable, deep injuries with a hollow-bore needle in a vein or artery of a male HCW corresponded to a higher risk of 6.669%.

#### Test sensitivity and specificity

Test sensitivities were derived from knowledge of the time interval from HCV exposure to: (1) the appearance of anti-HCV antibodies with third-generation assays; (2) the elevation of ALT; and (3) HCV RNA detection (table 1). The mean interval to the appearance of anti-HCV antibodies was previously estimated at 66 days^24^ to 82 days.^25^ ^26^ Mosley *et al* reported that the median latency period to an ALT elevation of >90 IU/l was 46 days.^27^ The interval between ALT elevation and anti-HCV antibody appearance is generally 1–2 weeks.^28^ The interval from HCV exposure to HCV RNA detection was reported to be 1–2 weeks^29^ with a mean of 10 days.^24^ In this analysis, a normal distribution was chosen to model the distribution of the times to the appearance of anti-HCV antibodies and HCV RNA. An exponential function was chosen to model the time to ALT elevation, so that the cumulative probability of this elevation at month 6 was <1, taking into account the possible fluctuation of ALT and the possible absence of detection of ALT elevation in some subjects. The derived sensitivities increased from 10.2% (range, 3.3–18.8%) at 1 month to 100% at 6 months for anti-HCV antibodies, from 36.8% (36.8–44.3%) to 93.6% (93.6–100%) for ALT, and were 100% for HCV RNA detection.

**Table 1:** Time to appearance of anti-HCV antibodies, ALT elevation and HCV RNA detection

Marker	Estimates	Model in baseline analysis	Models in sensitivity analysis
Anti-HCV antibodies	m = 66 days (SD = 28);^24^ 82 days (range, 54–192)^25^	Normal distribution (m = 66; SD = 28)	Normal distribution (m = 49; SD = 21) or Normal distribution (m = 82; SD = 28)
ALT	Median at 46 days;^27^ 1–2 weeks before HCV seroconversion^28^	Exponential distribution (λ* = 0.015)	Exponential distribution (λ = 1/52) or Exponential distribution (λ = 1/59)
HCV RNA	m = 10 days;^24^ 1–2 weeks^29^	Normal distribution (m = 10; SD = 3.5)	Normal distribution (m = 7; SD = 3.5) or Normal distribution (m = 14; SD = 7)

*λ is the parameter of the exponential distribution which is equal to ln(2) divided by the median time of elevation of ALT, which was set at 46 days in the base case analysis.^27^

ALT, alanine transaminase; HCV, hepatitis C virus; m, mean.

Specificities of 100% were assumed for anti-HCV antibody^30^^–^32 and HCV RNA detection.^33^ The specificity of the assay for ALT was derived from a study conducted in a population of Japanese male bank employees.^34^ In that study, the specificity of elevated ALT (>39 IU/l) was estimated at 86.3%.

#### Acute hepatitis C treatment efficacy

The French Haute Autorité en Santé (HAS) recommended treating acute hepatitis C with standard IFN or pegylated interferon monotherapy (PEG-IFN) (http://www.has-sante.fr/portail/upload/docs/application/pdf/06-072_hepat-c_internet_sans_liste.pdf).^35^ ^36^ Recently, a randomised trial assessing the efficacy and timing of PEG-IFN for treatment of acute hepatitis C^36^ has shown higher response rates with starting therapy at weeks 8 or 12 versus week 20. Moreover, another study assessing the efficacy, safety and optimal duration of PEG-IFN treatment^22^ has shown higher response rates with the 24 weeks regimen. These studies have been used in our modelling to set the timing and duration of therapy, and to calculate end of treatment response (ETR) and SVR rate (table 2). Moreover, we assumed that 26% of the subjects who seroconvert would spontaneously clear HCV by the end of the third month, 26% by the end of the fourth month, and 30% by the end of the sixth month.^36^

**Table 2:** Proportion of end of treatment response (ETR) and sustained viral response (SVR) with pegylated interferon during 6 months, according to timing of therapy after exposure^36^

Timing of therapy	ETR (%)	SVR (%)
Month 3 after exposure	95.3	93.2
Month 4 after exposure	91.8*	84.9*
Month 6 after exposure	84.8*	68.3*

*In the study of Kamal *et al*,^36^ ETR and SVR were observed for patients starting therapy at month 3 and month 5. We extrapolated ETR and SVR for patients starting therapy at month 4 and month 6 from these data according to a linear function.

#### Health-related quality of life

The quality of life of the HCWs probably deteriorates during the period of waiting for the detection tests to confirm or refute transmission, because of the anxiety generated by the fear of being infected, and was different among different strategies. In addition, in those in whom HCV transmission occurred, life expectancy and health-related quality of life were different when compared with those in whom HCV transmission did not occur.

As far as the quality of life of HCWs during the period of waiting for the detection test is concerned, we first assumed that the longer this period was, the more the quality of life will deteriorate. The literature does not include any estimates of the impact of the anxiety generated by the fear of being infected on the quality of life. Rodger *et al*^37^ examined the effect of diagnosis of HCV infection on the quality of life of a cohort of patients hospitalised with acute hepatitis 25 years ago. From this study, Thein *et al* estimated the utilities (ie, recommended health-related quality of life measure) associated with undiagnosed asymptomatic CHC.^38^ On the basis of these results, the utility we assigned during the month after exposure to HCWs waiting to be informed of their HCV status (ie, with undiagnosed hepatitis C) was 0.86. We assumed that this figure would increase by 0.04 for each month of waiting, and would reach 0.98 at 3 months after HCV exposure. We also assumed that utilities would be higher in HCWs sustaining injuries carrying a low risk of HCV transmission (0.90 at 1 month, 0.94 at month 2 and 0.98 thereafter), and lower in those sustaining injuries carrying a high risk (0.82 at 1 month, 0.86 at month 2, 0.90 at month 3, 0.94 at month 4 and 0.98 thereafter). For HCWs with diagnosed hepatitis C, the utility was assumed to be 0.77. HCWs with a negative HCV RNA test were assigned a utility of 1.

Utilities during and after acute hepatitis C treatment with PEG-IFN were based on the study of Thein *et al*^38^: 0.82 during the first 2 months, 0.81 during the following 2 months, and 0.79 during the last 2 months; utilities attributed to ETR and SVR were 0.80 and 0.87, respectively. Average discounted quality-adjusted life expectancy of a 42-year-old patient with CHC and without CHC (ie, a healthy person) was at 12.44 and 21.77 QALYs, respectively.^23^ Of note, the latest estimate matched the discounted-adjusted life expectancy of a 42-year-old healthy person in France (ie, 34 undiscounted years) (http://www.who.int/whosis/database/life_tables/life_tables.cfm).

#### Costs

The costs of screening tests, outpatient visits, treatment of acute hepatitis C, and treatment and follow-up of CHC for non-responders and relapsers to early treatment in the acute phase were taken into account in this analysis. For screening tests, the following unit costs were obtained from the French Nomenclature des Actes de Biologie Médicale (Nomenclature of medical biology acts) (available at http://www.ameli.fr/professionnels-de-sante/directeurs-d-etablissements-de-sante/index_savoie.php): €18.90 for anti-HCV antibody screening (third-generation ELISA), €5.94 for ALT measurement and €48.60 for HCV RNA detection. We assumed that HCWs attended an outpatient visit at the time of exposure, during follow-up when the HCV RNA was detected, before acute hepatitis C treatment initiation, during treatment (at 1 month, 3 months and at the end of treatment) and 6 months after the end of treatment for ETR and SVR. Doctors’ fees for an outpatient consultation were obtained from the French Nomenclature Générale des actes professionnels (General nomenclature for professional acts) and estimated at €26. Costs of 6 months PEG-IFN therapy for acute hepatitis C were calculated as the average cost of a treatment schedule between PEG-IFN alpha-2a and PEG-IFN alpha-2b (1.5 μg/kg per week): €5841.51. Total discounted lifetime costs of treatment and follow-up of CHC were from the study of San Miguel *et al* (ie, €26 000, with costs inflated to 2006 Euros.^23^

### Sensitivity analysis

First, we considered a low (0.009%) and high (6.669%) risk of HCV transmission after occupational exposure compared with the average of 0.5%. Next, we evaluated the implications of alternative assumptions in areas where we lacked primary data. We varied the distribution of the appearance of anti-HCV antibodies, ALT elevation and HCV RNA detection (table 1). We varied HCWs’ monthly quality of life while waiting for their HCV status over a wide range. We varied the proportion of the subjects who spontaneously clear HCV during the acute phase: between 20 and 30% at month 3 and 4, and between 26 and 35% at month 6. Finally, we varied the discounted cost of treatment and follow-up of CHC between €13 000 and €52 00.

### Software

The model was analysed with TreeAge Pro 2006 software (Williamstown, Massachusetts, USA).

## RESULTS

### Baseline analysis

Assuming the baseline risk of HCV transmission after a percutaneous injury, table 3 shows for a cohort of 7300 HCWs percutaneously exposed to HCV-positive source patients each year in France and for each studied strategy, the direct medical costs of follow-up, the mean time to HCV diagnosis, the number of patients who developed CHC and the number of CHC cases avoided by early therapy. The highest direct medical costs were obtained with the French strategy (€1 324 220 ) and the lowest with the baseline-US strategy (€924 180). The times to HCV infection diagnosis ranged from 1.0 for the alternative-US strategy to 6.0 months for the baseline-US strategy. Thus, the baseline-US strategy was the cheapest but was associated with the longest time to HCV infection diagnosis. In contrast, the alternative-US strategy was one of the most expensive but was associated with the shortest time to diagnosis. Moreover, it was associated with the lowest number of patients with CHC (1.9 vs 7.9 for the baseline-US strategy) and the highest number of CHC avoided by acute HCV treatment (25.1 vs 17.7 for the baseline-US strategy).

**Table 3:** Direct medical costs, mean time to HCV diagnosis, number of patients who developed CHC and number of CHC cases avoided by early therapy, associated with different follow-up strategies in 7300 French HCWs occupationally exposed to HCV

Strategy	Costs* (€)	Mean time to HCV diagnosis† (months)	No of CHC cases	No of CHC cases avoided by acute HCV treatment
French strategy based on anti-HCV antibody and ALT monitoring (strategy 1)	1 324 220	2.2	2.0	24.9
European strategy based on ALT monitoring (strategy 2)	1 132 230	2.0	2.1	24.9
Baseline-US strategy based on anti-HCV antibody and ALT monitoring (strategy 3)	924 180	6.0	7.9	17.7
Alternative-US strategy based on HCV RNA testing (strategy 4)	1 303 050	1.0	1.9	25.1

*Estimated costs from exposure to death.

†Mean time from exposure to HCV diagnosis.

ALT, alanine transaminase; CHC, chronic hepatitis C; HCV, hepatitis C virus; HCW, healthcare worker.

Table 4 shows the incremental costs, utilities and incremental C/E ratio for the baseline HCV transmission risk. Compared with the alternative-US strategy, the French strategy was associated with higher costs and lower utilities, and the European strategy with a higher incremental C/E ratio. Compared with the baseline-US strategy, the alternative-US strategy was associated with an incremental C/E ratio of €2020 per QALY saved.

**Table 4:** Costs, QALYs and C/E ratios associated with four follow-up strategies for HCWs occupationally exposed to HCV according to average risk of HCV transmission

Strategy	Costs per HCW (€)	QALYs	Incremental C/E ratio
Baseline-US	126.60	23.2292	
European	155.10	23.2409	Weakly dominated*
Alternative-US	178.50	23.2549	€2020
French	181.40	23.2396	Strongly dominated†

*A weakly dominated strategy a higher incremental C/E ratio than that of a more effective alternative strategy—that is, the incremental C/E ratio of the European strategy vs the baseline-US is higher than the incremental C/E ratio of the alternative-US strategy vs the European strategy.

†A strongly dominated strategy indicates a higher cost than that of a more effective alternative strategy—that is, the French strategy is associated with a higher cost and a lower utility compared with the alternative-US strategy.

C/E, cost-effectiveness; HCV, hepatitis C virus; HCW, healthcare worker; QALY, quality-adjusted life year.

### Sensitivity analysis

Table 5 shows the incremental costs, utilities and incremental C/E ratio for low and high HCV transmission risk. When we considered patients at low risk of HCV transmission, the C/E ratio of the alternative-US strategy compared with the baseline-US strategy was at €6102 per QALY saved. When the risk of HCV transmission was high, the baseline-US strategy was no longer the cheapest strategy because a higher number of CHC cases, which are associated with high costs, occurred with this strategy than with other strategies. The European strategy became the cheapest strategy, and the C/E ratio of the alternative-US strategy compared with the European strategy was at €695 per QALY saved.

**Table 5:** Costs, QALYs and C/E ratios associated with four follow-up strategies for HCWs occupationally exposed to HCV according to low and high risk of HCV transmission

Strategy	Costs per HCW (€)	QALYs	Incremental C/E ratio
Low HCV transmission risk			
Baseline-US	77.70	23.2498	
European	125.60	23.2551	Weakly dominated*
Alternative-US	149.70	23.2616	€6102
French	152.00	23.2501	Strongly dominated†
High HCV transmission risk			
European	525.00	23.1870	
Alternative-US	540.30	23.2090	€695
French	550.70	23.1850	Strongly dominant†
Baseline-US	740.60	23.0830	Strongly dominant†

*A weakly dominated strategy indicates a higher incremental C/E ratio than that of a more effective alternative strategy—that is, i.e. the incremental C/E ratio of the European strategy vs the baseline-US is higher than the incremental C/E ratio of the alternative-US strategy vs the European strategy (with low HCV transmission risk).

†A strongly dominated strategy indicates a higher cost than that of a more effective alternative strategy—that is, the French strategy is associated with a higher cost and a lower utility compared with the alternative-US strategy (with low HCV transmission risk), and the French and baseline-US strategies are associated with higher costs and lower utilities compared with the European strategy (with high HCV transmission risk).

C/E, cost-effectiveness; HCV, hepatitis C virus; HCW, healthcare worker; QALY, quality-adjusted life year.

When we varied the health-related quality of life of HCWs, while waiting for their HCV status and we assigned a utility of 0.99 per month (vs 0.86 at month 1, 0.90 at month 2, 0.94 at month 3, and 0.98 thereafter in the base case analysis), first the European strategy was no longer dominant and when compared with the baseline-US strategy it was associated with a C/E ratio of €3314 per QALY saved. Compared with the European strategy, the alternative-US strategy was still associated with a reasonable C/E ratio of €8069 per QALY saved. When we assumed that waiting for HCV status had no impact on the HCW’s quality of life (utilities at 1), compared with the baseline-US strategy the C/E ratio of the European strategy was estimated at €3958 per QALY saved. Compared with the European strategy, the C/E ratio of the alternative-US strategy was estimated at €234 000 per QALY saved.

Results were robust to variations in: (1) distributions used to model the appearance of anti-HCV antibodies, ALT elevation and HCV RNA detection; (2) in the proportion of the subjects who spontaneously cleared HCV during the acute phase; and (3) the cost of treatment and follow-up of CHC.

## DISCUSSION

In this study, the cost and effectiveness of four strategies for follow-up after occupational exposure to HCV were compared. Although more expensive, a strategy based on early HCV RNA testing was found to be associated with a reasonable C/E ratio compared with strategies based on ALT detection and/or anti-HCV antibody detection. Whatever the risk of HCV transmission considered, the C/E ratio of this strategy was lower than the ratio of US$50 000 (about €37 000, £29 000) per QALY for dialysis for end-stage renal disease, often described as a benchmark for C/E.^39^ Moreover, the C/E of this strategy was less than the Gross Domestic Product (GDP) per capita of France in 2006 (ie, €30 000), designed as the threshold for considering interventions as “very cost-effective”.^40^

Our results are first related to the fact that HCV RNA testing may lead to earlier detection of acute hepatitis C when compared with other strategies, and therefore to earlier initiation of anti-HCV therapy. We tested the impact of earlier diagnosis on the response to treatment, using data from the study of Kamal *et al*.^36^ This study showed that PEG-IFN therapy induces high SVR rates and prevents CHC, and that SVR varied according to timing of therapy.^36^ Our analysis suggested that the alternative-US strategy would lead to a slightly lower number of CHC cases than the French and European strategies. Moreover, it suggested that the alternative-US strategy would lead to a much lower number of CHC cases than the baseline-US strategy (1.9 vs 7.9 CHC among 7300 HCWs percutaneously exposed to anti-HCV antibody-positive source patients).

Our results are also related to the impact of occupational exposure on the health-related quality of life of HCWs. We found that with the strategy based on early HCV RNA testing, the absence of HCV transmission to HCWs could be confirmed earlier than with the other strategies. As a result, HCW quality of life deteriorated less, because the period of waiting for the detection tests to confirm or refute transmission was shorter. One of this study’s main limitations was the lack of data on the quality of life of HCWs awaiting the results of detection tests. We used utilities associated with undiagnosed asymptomatic CHC as a substitute for estimating the quality of life of an HCW exposed to an HCV-infected source patient. We noted with interest that our conclusions were robust over a wide range of values for quality of life of HCWs during sensitivity analysis. In particular, an early HCV RNA testing strategy was associated with a reasonable C/E ratio even when we considered that the quality of life of an HCW was very moderately modified while they were waiting for their HCV status after an exposure. The HCV RNA testing strategy was not more cost-effective only when we assumed that the quality of life of an HCW was not modified at all while they were waiting for their HCV status, which is unlikely.

In the present analysis, we did not take into account the fact that a large proportion of HCWs are lost to follow-up during the period after occupational exposure. However, the longer the follow-up period until the detection of HCV transmission, the greater the probability of loss to follow-up. The proportion of patients lost to follow-up is therefore probably lower with a strategy based on early HCV RNA testing. Again, if we had incorporated losses to follow-up in our analysis, early HCV RNA testing would probably have been even more cost-effective.

Finally, we did not consider the costs of the time lost by HCWs who had to attend outpatient visits. However, all these costs weigh heavier for Strategies 1, 2 and 3 than for Strategy 4. If we had included them in the analyses, HCV RNA testing would have been even more cost-effective and perhaps even cost-saving.

In conclusion, in HCWs exposed to HCV, a strategy based on HCV RNA testing leads to earlier detection of HCV transmission. It is therefore proposed that anti-HCV therapy should take place earlier in these patients than with other strategies, which would result in a lower risk of progression to CHC. Moreover, a strategy based on HCV RNA testing leads to earlier confirmation of the absence of HCV transmission than other strategies, and therefore shortens the period during which the HCW’s quality of life deteriorates. We found that although the HCV RNA testing strategy is more expensive than the other strategies examined, it is reasonably cost-effective for all the risks of HCV transmission considered. We therefore recommend its use for occupational HCV detection.
